# Quantitative redox proteomics revealed molecular mechanisms of salt tolerance in the roots of sugar beet monomeric addition line M14

**DOI:** 10.1186/s40529-022-00337-w

**Published:** 2022-03-05

**Authors:** He Liu, Xiaoxue Du, Jialin Zhang, Jinna Li, Sixue Chen, Huizi Duanmu, Haiying Li

**Affiliations:** 1 Key Laboratory of Molecular Biology of Heilongjiang Province, College of Life Sciences, Heilongjiang University, Harbin, 150080 China; 2 Engineering Research Center of Agricultural Microbiology Technology, Ministry of Education, Heilongjiang University, Harbin, 150080 China; 3 Heilongjiang Provincial Key Laboratory of Ecological Restoration and Resource Utilization for Cold Region, School of Life Sciences, Heilongjiang University, Harbin, 150080 China; 4 Proteomics and Mass Spectrometry, Interdisciplinary Center for Biotechnology Research, University of Florida, Gainesville, FL 32610 USA; 5 Department of Biology, Genetics Institute, Plant Molecular and Cellular Biology Program, University of Florida, Gainesville, FL 32610 USA

**Keywords:** Sugar beet M14, Redox proteomics, iodoTMTRAQ, Salt stress, Molecular mechanisms

## Abstract

**Background:**

Salt stress is often associated with excessive production of reactive oxygen species (ROS). Oxidative stress caused by the accumulation of ROS is a major factor that negatively affects crop growth and yield. Root is the primary organ that senses and transmits the salt stress signal to the whole plant. How oxidative stress affect redox sensitive proteins in the roots is not known.

**Results:**

In this study, the redox proteome of sugar beet M14 roots under salt stress was investigated. Using iTRAQ reporters, we determined that salt stress caused significant changes in the abundance of many proteins (2305 at 20 min salt stress and 2663 at 10 min salt stress). Using iodoTMT reporters, a total of 95 redox proteins were determined to be responsive to salt stress after normalizing again total protein level changes. Notably, most of the differential redox proteins were involved in metabolism, ROS homeostasis, and stress and defense, while a small number play a role in transport, biosynthesis, signal transduction, transcription and photosynthesis. Transcription levels of 14 genes encoding the identified redox proteins were analyzed using qRT-PCR. All the genes were induced by salt stress at the transcriptional level.

**Conclusions:**

Based on the redox proteomics results, we construct a map of the regulatory network of M14 root redox proteins in response to salt stress. This study further refines the molecular mechanism of salt resistance at the level of protein redox regulation.

**Supplementary Information:**

The online version contains supplementary material available at 10.1186/s40529-022-00337-w.

## Background

Soil salinity is a worldwide ecological and resource problem, which has a negative impact on crop production. Statistics from the International Food and Agriculture Organization shows that around 800 million hectares of land worldwide are affected by salinity (FAO 2008). Growth and productivity of most glycophytes are compromised by salt stress (Slama et al. 2015). Under salt stress, besides osmotic stress and ion toxicity, ROS overaccumulation is a secondary stress that further impairs plant performance. (Liu et al. 2021; Yang and Guo 2018). Oxidative stress is caused by high levels of ROS in plant cells (Mittler 2002). Proteins are the main target molecules to sustain oxidative damage (Pena et al*.* 2012). ROS have been shown to mediate post-translational modifications (PTMs) of proteins by oxidation of cysteine residues (Navrot et al. 2011). Specifically, cysteine free sulfhydryl group (–SH) may be oxidized to reversible cysteine sulfenic acid (–SOH), disulfide bonds (S–S), nitrosylation (SNO) and glutathionylation (–SSG), as well as irreversible cysteine sulfonic acid (SO_2_H) and sulfonic acid (SO_3_H). Redox homeostasis is maintained by regulating protein microenvironment to alleviate the effect of salt stresses. Currently, most plant redox proteomics studies have focused on the reversible oxidative modification of cysteines (Menon and Goswami 2007; Diaz-Vivancos 2015). In addition, the ratios of ascorbate (AsA) to dehydroascorbate or GSH to GSSG were found to be important markers of plant cellular redox state under stress conditions (Aliyeva et al. 2020; Hasanuzzaman et al. 2019; Navrot et al. 2011).

There are several redox proteomics techniques for studying protein redox changes under stress conditions. Initially, gel-based proteomics using thiol-specific reagents was widely utilized to label reduced thiols, and then using two-dimensional electrophoresis (2DE) to separate and identify differentially labelled proteins (Alvarez et al. 2009; Nogueira et al. 2012; Wang et al. 2012). A cysteine targeting approach has provided a high-throughput platform for studying plant redox proteomics. Isotope-encoded affinity tags (ICAT) (Fu et al. 2008), OxICAT (Leichert et al. 2008), multiple reaction monitoring (MRM) (Held et al. 2010), thioredoxin affinity chromatography, and several other as well (Picotti and Aebersold 2012). In recent years, iodoacetyl tandem mass label (iodoTMT) (Pan et al. 2014; Qu et al. 2014) high-throughput screening methods have become common. Isobaric tags for relative and absolute quantification (iTRAQ) and their modifications such as oxiTRAQ (Liu et al. 2014), cysTRAQ (Zhang et al. 2016) have been developed and utilized. Although iodoTMT is able to quantify oxidatively modified proteins, it cannot simultaneously quantify protein abundance or accurately determine changes in protein redox levels without considering total protein level changes, thus it may lead to misleading results. iodoTMTRAQ dual-labelling technology can simultaneously detect changes in Cys redox levels and protein expression abundance, providing an accurate determination of changes in protein redox levels (Yin et al. 2017). It has been shown that 47 potential redox-regulated proteins were identified in *Arabidopsis* suspension cells by iodoTMTRAQ double-labelling technology (Yin et al. 2017). Using the same approach, 35 potentially protective cellular proteins regulated by SNO in response to the bacterial peptide inducer flg22 were identified (Lawrence et al. 2020).

Sugar beet M14 monosomic addition line was obtained from an interspecies cross between cultivated sugar beet (*Beta vulgaris*) and wild *B. corolliflora*. It contains 18 normal chromosomes of sugar beet and chromosome 9 of *B. corolliflorais*, and shows stress tolerance (Guo et al. 2001). Comparative proteomic and transcriptomic analyses between the M14 and *B. vulgaris* identified 71 proteins that were differentially expressed (Li et al. 2009; Zhu et al. 2009). In recent years, an increasing number of M14 proteomic studies have been reported. Yang et al. (2012) used 2DE to analyze the proteomics of M14 roots and leaves under salt stress, and found uniquely expressed proteins in roots and leaves. Furthermore, they reported 75 differentially expressed proteins in M14 leaves and 43 differentially expressed proteins in roots using quantitative proteomics (Yang et al. 2013). A couple of years later, Li et al. (2015) used iTRAQ 2D LC–MS/MS technology to perform quantitative proteomic analysis of sugar beet membrane proteins under salt stress to identify significantly altered membrane proteins and determine their possible relevance to salt tolerance. Similarly, phosphorylation proteomics studies were carried out in the M14 (Yu et al. 2016). Recently, redox proteomics of sugar beet leaves under salt stress using iodoTMTRAQ dual-labelled quantitative proteomics approach has also been reported (Li et al. 2021), which has helped to understand the mechanisms of salt tolerance in sugar beet M14. Although various studies have been carried out, redox proteomics of M14 roots has not been reported, and a comprehensive and in-depth exploration of its root redox proteome is necessary.

In this study, we used the iodoTMTRAQ dual-labelling technology to investigate changes in redox proteins and total protein levels in a single experiment. This study revealed different functions of the differential redox proteins and the different pathways involved. Combined with the analysis of the changes at the transcript level of the genes encoding the differential proteins, it has provided insight into the physiological response strategies and molecular regulatory mechanisms of salt stress tolerance in sugar beet M14. The knowledge forms a theoretical basis for the use of genetic engineering and/or molecular breeding tools for improving crop resilience.

## Materials and methods

### Plant material, salt stress treatment and physiological indicators measurement

The M14 seeds were soaked in water for 4 h, disinfected with 70% ethanol for 1 min, soaked for 15 min using 0.1% HgCl_2_, treated with TMTD (1:500) for 12 h and rinsed in water. The treated seeds were sown in white porcelain trays lined with vermiculite and incubated at 25 °C/20 °C (day/night) in a light chamber with a light intensity of 450 µmol m^−2^ s^−1^, a light duration of 14 h and relative humidity of 65%. After 7 days, the seedlings were transferred into a half strength Hoagland’s nutrient solution (Cherki et al. 2002) for hydroponics, and then treated with salt stress when the fifth real leaf emerged. *BvM14* seedlings were treated with 0 mM NaCl as a control and the final concentration of NaCl was added to the nutrient solution up to 200 mM and 400 mM as salt stress treatments. Root samples from three individual plants (each as a biological replicate) were snap frozen in liquid nitrogen after harvesting and stored at − 80 °C till further use. Free sulfhydryl group of cysteine, AsA and GSH content was measured following a manufacturer protocol (Comin Biotechnologies, Suzhou, China). Three biological repeats were used for each analysis.

### Protein sample preparation

The root samples were ground to a powder in liquid nitrogen with cysteine alkylation reagent *N*-ethylmaleimide (NEM), and the total protein was extracted by phenol extraction. In particular, equilibrated phenol (pH = 7.8) was added to the samples contained in the tubes, mixed thoroughly and then a phenol extraction buffer (900 mM sucrose, 100 mM Tris–HCl (pH8.8), 1 mM PMSF, 20 mM *N*-ethylmaleimide (NEM), 10 mM EDTA) was added, mixed well and centrifuged. To the protein fraction, 5 times the volume of 100% methanol containing 0.1 M ammonium acetate was added. The mixture was incubated overnight at − 20 °C. After centrifugation at 20,000 r/min for 20 min at 4 °C, the pellet was collected and washed with pre-cooled 80% and 100% acetone respectively. A protein lysis buffer (0.5% SDS, 6 M Urea, 30 mM Tris–HCl, pH 8.5) was added to solubilize the pellet. Protein concentration was determined using a bicinchoninic acid (BCA) kit according to the manufacturer's instructions (TAKARA, Beijing China).

### iodoTMTRAQ labeling, strong cation exchange fraction and LC–MS/MS

The reversibly oxidized cysteine thiols in the protein samples were firstly reduced for reverse labelling by incubating the protein samples with 5 mM of tris(2-carboxyethyl) phosphine at 50 °C for 1 h. We labelled control samples with 126, 128 and 130 TMT reagents for 0, 10 and 20 min and salt-treated samples with 127, 129 and 131 reagents, respectively. The labelling was performed for 2 h at 37 °C in the dark, followed by quenching with 0.5 M DTT for 15 min at 37 °C in the dark. Trypsin (sequencing grade, Promega, Madison) was added at an enzyme to protein ratio of 1:50 (w/w) and digested overnight at 37 °C (Parker et al. 2012). Peptides were cleaned up using a C18 desalting column (The Nest Group Inc., Southborough, MA) and lyophilized to dryness. The C18 cleaned peptides were labelled with iTRAQ reagent according to the manufacturer's protocol (AB Sciex Inc., Framingham, MA, USA). The control samples at 0, 10 and 20 min were labelled with reporter labels 113, 115 and 117, respectively, while treatment samples were labelled with reporter labels 114, 116 and 119. Labelling was maintained at 37 °C for 2 h and labelled peptides were desalted according to published procedures (Parker et al. 2012; Yu et al. 2016). LC–MS/MS was connected to an Easy-nLC 1000 on a Q-Exactive Plus MS/MS system (Thermo Fisher Scientific, Bremen, Germany). Tandem mass spectrometry was performed following the method of Yu et al. (2016).

### Bioinformatics analysis

Data analysis for peptide MS2 spectra was performed by Thermo Fisher’s Proteome Discoverer 2.1, searching the combined Sugar Beet Protein Database and the Green Plant Protein Database from NCBI (with a total of 6255663 ntries). Oxidatively modified protein and total protein data were normalized to the 126 tag in the iodoTMT reporter and the 113 tag in the iTRAQ reporter, respectively. The control group was used as a criterion to screen peptides with P-values < 0.05, while fold-change analysis was performed to select peptides with fold-change > 1.2 and < 0.8 as significant peptides on the redox level and protein abundance level. The full sequences of the differential proteins were queried in the Protein Data Bank of NCBI (http://www.ncbi.nlm.nih.gov/protein/), UniProt database (http://www.ebi.uniprot.org/) using Gi numbers. Functional annotations of redox proteins were obtained using GO (http://geneontology.org/) and combined with relevant literature, and KEGG pathways (https://www.kegg.jp/). Subcellular localization was predicted using online analysis tools (YLoc, LocTree3, ngLOC, TargetP). The redox protein network of sugar beet M14 roots under salt stress was mapped using Adobe Illustrator 2021. Physiological and biochemical index data and qRT-PCR results were analyzed and data processed using GraphPad Prime 6 software. Significant differences were analyzed with * indicating P < 0.05 and ** indicating P < 0.01.

### qRT-PCR

The genes encoding differential redox proteins were selected for real-time quantitative PCR (qRT-PCR) in order to test possible correlation between the transcription level and protein level under 200 mM and 400 mM NaCl treatment conditions. A total of 14 differential redox proteins involved in ROS homeostasis and signal transduction, and differential redox proteins in roots and leaves were selected. Total RNA from sugar beet M14 roots was extracted with Trizol, cDNA templates were obtained using a reverse transcription kit (TAKARA) and qRT-PCR was performed using the SYBR dye method with the 18S rRNA reference gene (Zhang et al. 2015). Each reaction consisted of three biological replicates and three technical replicates. The relative expression levels of the target genes were calculated by normalizing against an internal standard 18S by the − ΔΔCt method.

## Result

### Changes of cysteine free sulfhydryl, AsA and GSH contents in roots of sugar beet M14 treated with salt stress

**Fig. 1:**
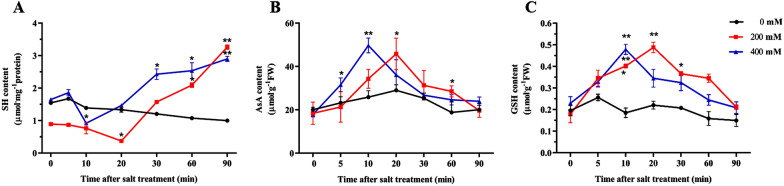
Temporal changes in cysteine free sulfhydryl, AsA, and GSH contents in *BvM14* roots under salt stress. **A** Cysteine free sulfhydryl content under 200 mM and 400 mM NaCl stress. **B** ASA content under 200 mM and 400 mM NaCl stress. **C** GSH content under 200 mM and 400 mM NaCl stress. These values are the means of three biological replicates from different samples with standard errors. *p < 0.05; **p < 0.01

The changes in cysteine free sulfhydryl, ASA and GSH over a 90 min time-course of treatment with different salt concentrations are shown in Fig. 1. Under control conditions, the lowest levels of cysteine free sulfhydryl were reached at 20 min (200 mM NaCl) and 10 min (400 mM NaCl) in response to the salt stress (Fig. 1A). Excessive accumulation of ROS in plants induced by salt stress prompted oxidative modification of cysteine sulfhydryl groups and a decrease in free sulfhydryl content, indicating the highest level of oxidative modification of proteins at this time. Further studies revealed that the levels of AsA and GSH in the sugar beet M14 roots remained stable. Their levels peaked at 20 min (200 mM NaCl) and 10 min (400 mM NaCl) under salt stress (Fig. 1B, C). The results clearly indicate that salt stress caused significant changes in cellular redox status as early as 10 min after treatment. Based on these results, we selected samples collected at 200 mM NaCl for 20 min and 400 mM NaCl for 10 min for iodoTMTARQ-based redox proteomics studies.

### LC–MS/MS analyses of root proteins and redox proteins in BvM14 response to salt stress

**Fig. 2:**
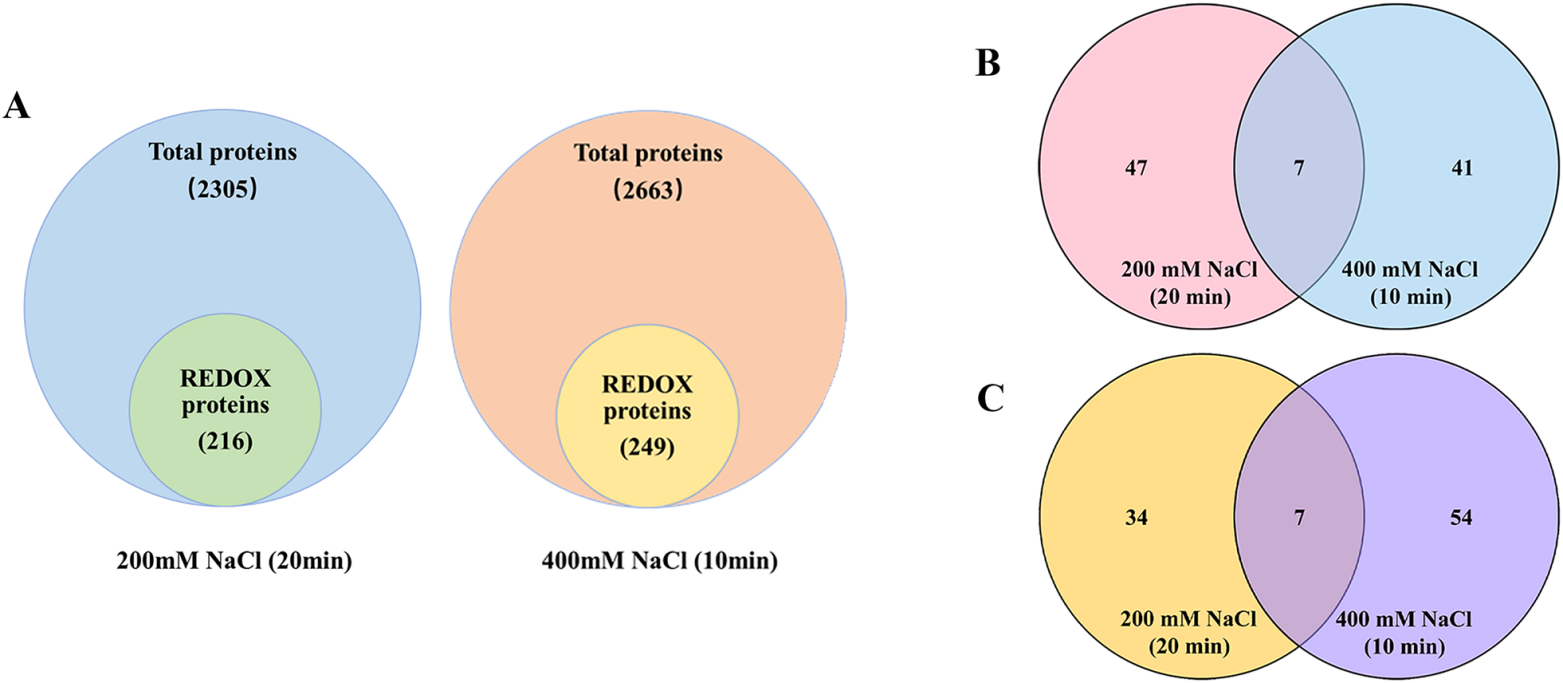
Visualization of redox protein profile data from *BvM14* roots under salt stress. **A** iTRAQ-labeled total protein and iodoTMT-labeled redox protein of *BvM14* under 200 mM and 400 mM NaCl stress. **B** Significant changes in protein redox levels in *BvM14* roots under salt stress. **C** Comparison of the number of differential redox proteins identified under 200 mM NaCl and 400 mM NaCl treatments

LC–MS/MS quantitative analysis identified 2305 proteins (20 min) (Additional file 2: Table S1) and 2663 proteins (10 min) (Additional file 3: Table S2) with iTRAQ tags. There were 462 (20 min) and 279 (10 min) proteins that showed significant changes in protein abundance. A total of 260 (20 min) (Additional file 4: Table S3) and 249 (10 min) (Additional file 5: Table S4) proteins with iodoTMT tags were identified as having significant changes in redox levels. Among them, 42 (20 min) and 63 (10 min) proteins screened by bioinformatic analysis showed significant changes in redox levels, while 41 (20 min) and 61 (10 min) of these proteins did not exhibit significant changes in protein abundance (Fig. 2A). A total of 95 redox proteins were identified under 200 mM and 400 mM NaCl stress (Table 1). There was also variable expression among the identified redox proteins, with 54 proteins oxidized (FC > 1.2) and 48 proteins reduced or irreversibly oxidized (FC < 0.8) (Fig. 2B). Among them, there were 34 unique redox proteins under 200 mM NaCl treatment and 54 unique redox proteins under 400 mM NaCl treatment (Fig. 2C). Notably, there were seven redox proteins under salt stress, three of which had the same total protein level and significantly increased oxidation levels. They were identified as proteasome subunit beta-6 type (PBA6), protein P21 (P21) and basic 7S globulin (Bg7s). Bioinformatic analysis indicated that these proteins are important oxidative sensors of root responses to salt stress in M14.

**Table 1:** A list of 95 differential redox proteins in *BvM14* roots between control and NaCl-treated groups

No.	Protein ID^a^	Description	Abbreviation	Sequence with modification^b^	Plant species	iodoTMT salt200/control ratio^c^	iodoTMT salt400/control ratio^d^	*p*-value	Protein location^e^
**Metabolism**									
*Carbohydrate metabolism*									
1	A0A2H5P1K5	6-Phosphogluconate dehydrogenase, decarboxylating	PGDH	IC^2^SYAQGMNILR	*Citrus unshiu*	–	1.38	0.05	Chloroplast
2	731322678	Beta-fructofuranosidase, soluble isoenzyme I	β-FFase	NWFC^4^TDQSR	*Beta vulgaris* subsp. vulgaris	–	0.76	0.03	Vacuole
3	Q41140	Pyrophosphate–fructose 6-phosphate 1-phosphotransferase subunit alpha	PFP1	SLYKPELPPC^10^LQGTTVR	*Ricinus communis*	–	0.74	0.03	Cytoplasm
4	1108966238	Sucrose synthase isoform X2	SUS	LLPDAVGTTC^10^GQR	*Beta vulgaris* subsp. vulgaris	–	0.64	0.05	Chloroplast
5	731323052	Probable fructokinase-4	FRK	LLLVTLGDQGC^11^R	*Beta vulgaris* subsp. vulgaris	0.75	–	0.04	Cytoplasm
6	A0A0S3T1M9	UDP-glucose 6-dehydrogenase	UGDH	VFDC^4^MQKPAFVFDGR	*Vigna angularis* var. angularis	–	1.30	0.02	Cytoplasm
7	731364471	Trypsin inhibitor BvTI	TI	NPELPC^6^PYYITR	*Beta vulgaris* subsp. vulgaris	0.30	–	0.04	Extracellular
8	731344067	Kunitz trypsin inhibitor 1-like	KTI	C^1^PYYSVVQSQDDR	*Beta vulgaris* subsp. vulgaris	–	1.50	0.01	Vacuole
9	731331165	Alpha-amylase/trypsin inhibitor	α-TI	ANGGC^5^NNAYNYSYSR	*Beta vulgaris* subsp. vulgaris	0.52	0.49	0.01	Extracellular
*Amino acid metabolism*									
10	731353768	Aspartate aminotransferase	AST	VASAQC^6^LSGTGSLR	*Beta vulgaris* subsp. vulgaris	–	1.20	0.03	Cytoplasm
11	A0A2P5X5J0	Aspartate aminotransferase	AST	IAAVQALSGTGAC^13^R	*Gossypium barbadense*	–	1.26	0.04	Cytoplasm
12	731351009	Aspartic proteinase A1-like	AP	VGEGPAAQC^9^ISGFTALDVPPPR	*Beta vulgaris* subsp. vulgaris	1.35	–	0.02	Vacuole
13	731353609	3-Hydroxyisobutyryl-CoA hydrolase-like protein 3, mitochondrial isoform X1	H2BCH	C^1^VLIESSSPR	*Beta vulgaris* subsp. vulgaris	–	1.21	0.01	Mitochondrial
14	A0A2I0XB93	Aspartate-semialdehyde dehydrogenase	ASDH	IRQDLSQEGNHGLDIFVC^18^GDQIR	*Dendrobium catenatum*	–	1.41	0.02	Cytoplasm
15	A0A0M3TGF7	Acetolactate synthase	ALS	C^1^GISDVFAYPGGASMEIHQALTR	*Poa annua*	–	1.33	0.03	Chloroplast
16	731325199	Serine hydroxymethyl transferase 4	SHMT	MLIC^4^GGSAYPR	*Beta vulgaris* subsp. vulgaris	0.59	–	0.04	Cytoplasm
17	731317741	LL-diaminopimelate aminotransferase, chloroplastic	DAPL	TELIFFC^7^SPNNPTGAAATR	*Beta vulgaris* subsp. vulgaris	–	0.80	0.05	Chloroplast
18	A0A0K9RN52	Glutamate-1-semialdehyde 2,1-aminomutase	GSAM	FVNSGTEAC^9^MGVLR	*Spinacia oleracea*	1.21	–	0.00	Chloroplast
*Other metabolism*									
19	A0A0B2RAS0	Proteasome subunit alpha type-5	PSAM5	FSYGEPMTVESTTQAIC^17^DLALR	*Glycine soja*	0.76	–	0.05	Nucleus
20	731363918	Proteasome subunit alpha type-5	PSAM5	FSYGEPMTVESTTQALC^17^DLALR	*Beta vulgaris* subsp. vulgaris	–	1.24	0.00	Nucleus
21	731361751	Proteasome subunit alpha type-5	PSAM5	FSYGEPMNVESTTQALC^17^DLALR	*Beta vulgaris* subsp. vulgaris	–	1.46	0.03	Nucleus
22	A0A287HDI6	Proteasome subunit beta type-6	PSAM6	QLTDNVYVC^9^R	*Hordeum vulgare* subsp. vulgare	1.23	1.57	0.05	Nucleus
23	M0UCJ4	ATP synthase subunit beta	ATPsny	VC^2^QVIGAVVDVR	*Musa acuminata* subsp. malaccensis	0.74	–	0.04	Mitochondrion
24	M8C108	ATP synthase subunit alpha, mitochondrial	ATPsny	MTNFC^5^TNFQVDEIGR	*Aegilops tauschii*	–	1.76	0.01	Mitochondrial
**ROS homeostasis**									
25	A0A287X935	Peroxidase	POD	ASVEAVC^7^PGVVSC^13^ADILAITAR	*Hordeum vulgare* subsp. vulgare	–	2.13	0.01	Extracellular
26	A0A2G9HTZ9	Peroxidase	POD	QAVEAQC^7^PGVVSC^13^SDILAIAAR	*Handroanthus impetiginosus*	–	2.05	0.01	Extracellular
27	A0A1S2YYJ3	Peroxidase	POD	SDLENAC^7^PSTVSC^13^ADILTLAAR	*Cicer arietinum*	–	1.70	0.01	Extracellular
28	A0A2G2WVY9	Peroxidase	POD	IKTMC^5^PGAAVSC^12^ADILALAAR	*Capsicum baccatum*	0.46	–	0.05	Extracellular
29	J3L3F3	Peroxidase	POD	LEAAC^5^PKTVSC^11^ADILALAAR	*Oryza brachyantha*	–	1.65	0.01	Extracellular
30	A0A0J8CS88	Peroxidase	POD	QC^2^PAGNAGANIVVPMDPISPTISDTAYYR	*Beta vulgaris* subsp. vulgaris	–	1.50	0.04	Extracellular
31	731316487	Peroxidase 4	POD4	TC^2^PQLFPTIR	*Beta vulgaris* subsp. vulgaris	–	0.56	0.01	Extracellular
32	731313635	Peroxidase 12	POD12	VVSC^4^ADITSLAAR	*Beta vulgaris* subsp. vulgaris	0.42	–	0.05	Extracellular
33	731313633	Peroxidase 12	POD12	VVSC^4^ADITTLAAR	*Beta vulgaris* subsp. vulgaris	–	0.69	0.04	Extracellular
34	731313639	Peroxidase 12	POD12	VVSC^4^ADLTALAAR	*Beta vulgaris* subsp. vulgaris	0.64	–	0.03	Vacuole
35	A0A0A9MG34	Peroxidase 72	POD72	AALEAAC^7^PSTVSC^13^ADILALTAR	*Arundo donax*	–	1.55	0.05	Extracellular
36	731337443	Peroxidase 72	POD72	AAVEQAC^7^PHTVSC^13^ADILALTAR	*Beta vulgaris* subsp. vulgaris	–	2.32	0.03	Extracellular
37	731331163	Protein P21	P21	TDNYC^5^C^6^NSGSC11GPTDYSR	*Beta vulgaris* subsp. vulgaris	4.09	1.44	0.02	Extracellular
38	A0A1S3TTL2	DSBA domain-containing protein	DSBA	NVGLEYC^7^MSGLTGNTIDSHR	*Vigna radiata* var. radiata	0.55	1.63	0.04	Chloroplast
39	731339890	EG45-like domain containing protein 2	EG45	VTDLC^5^DSC^8^AGDLNLSQEAFNVIADTR	*Beta vulgaris* subsp. vulgaris	–	0.44	0.00	Extracellular
40	731352762	EG45-like domain containing protein	EG45	VTC^3^VSGTNQGVPQPC^15^R	*Beta vulgaris* subsp. vulgaris	–	1.32	0.04	Extracellular
41	A0A0J8B2W2	Fe2OG dioxygenase domain-containing protein	Fe2OG	VAIYPEC^7^PNPELVR	*Beta vulgaris* subsp. vulgaris	–	0.59	0.02	Cytoplasm
42	M0RV51	Glutathione *S*-transferase DHAR2	GST	AAVGAPDVLGDC^12^PFSQR	*Musa acuminata* subsp. malaccensis	0.64	–	0.01	Cytoplasm
43	A0A199UJ48	3-Ketoacyl-CoA thiolase 2, peroxisomal	HT	IELFAQARDC^10^LLPMGITSENVAHR	*Ananas comosus*	–	1.45	0.00	Peroxisome
44	731355863	l-Ascorbate oxidase-like	AOX	QLGTPWADGTASISQC^16^PINPGETFTYR	*Beta vulgaris* subsp. vulgaris	0.51	1.58	0.01	Plasma Membrane
45	A0A151QMI1	Nitrate reductase [NADH] 2	NR	QSGALHVC^8^FEGAEDLPGGGGSKYGTSVTR	*Cajanus cajan*	–	1.54	0.00	Peroxisome
46	731357289	NADH dehydrogenase [ubiquinone] 1 alpha subcomplex subunit 8-B	NADH	C^1^VFSLLR	*Beta vulgaris* subsp. vulgaris	–	1.29	0.04	Mitochondrion
47	731359814	Peptide methionine sulfoxide reductase B5-like	MSR	FDSGC^5^GWPAFYEGLPGAITR	*Beta vulgaris* subsp. vulgaris	–	0.75	0.02	Cytoplasm
48	731312054	Cysteine protease RD19A	RD19A	LVSLSEQQLVDC^12^DHEC^16^DPEER	*Beta vulgaris* subsp. vulgaris	1.63	–	0.04	Vacuole
**Stress and defense**									
49	731330989	Probable polygalacturonase	PGs	VIDNFEYSAINC^12^R	*Beta vulgaris* subsp. vulgaris	1.5	–	0.04	Plasma Membrane
50	731338906	PLAT domain-containing protein 3	PITI	GPC^3^LNAPVC^9^AMR	*Beta vulgaris* subsp. vulgaris	–	1.26	0.01	Vacuole
51	A0A166FTZ6	Heat shock cognate 70 kDa protein	Hsp70	MDIC^4^SVHDVVLVGGSTR	*Daucus carota* subsp. sativus	–	1.21	0.05	Endoplasmic reticulum
52	Q9XFW7	Chitinase	–	FGFC^4^GSTDAYC^11^GEGC^15^R	*Beta vulgaris* subsp. vulgaris	2.05	–	0.01	Extracellular
53	731352263	Endochitinase EP3	EP3	VGYYTQYC^8^QQLGVSPGNNLR	*Beta vulgaris* subsp. vulgaris	–	0.65	0.02	Cell Wall
54	731352251	Endochitinase EP3	EP3	AINGGEC^7^GGGNTPAVNAR	*Beta vulgaris* subsp. vulgaris	–	0.45	0.00	Cell Wall
55	731352259	Endochitinase EP3	EP3	LEC^3^DGGNPATVNAR	*Beta vulgaris* subsp. vulgaris	0.71	–	0.01	Cell Wall
56	731329194	Pathogenesis-related protein PR-4	PR-4	NQYGWTAFC^9^GPAGPTGQASC^20^GR	*Beta vulgaris* subsp. vulgaris	1.64	–	0.01	Cytoplasm
57	731326017	Jasmonate-induced protein homolog	JIP	LDASHDESHC^10^PGAAAR	*Beta vulgaris* subsp. vulgaris	–	1.33	0.02	Cell wall
58	731332586	Jasmonate-induced protein homolog	JIP	LENSGNC^7^SYDVDYETR	*Beta vulgaris* subsp. vulgaris	0.36	–	0.04	Cell wall
59	731312253	Jasmonate-induced protein homolog	JIP	C^1^GPAAEFNNVNWTQVR	*Beta vulgaris* subsp. vulgaris	–	1.49	0.00	Cell wall
60	A0A2P4NB14	Flavonoid 3′,5′-methyltransferase	GIP	IESSLLSIGDGITLC^15^R	*Quercus suber*	–	1.33	0.02	Cytoplasm
61	731357526	lysM domain-containing GPI-anchored protein 2		STC^3^AYVGYNR	*Beta vulgaris* subsp. vulgaris	0.53	–	0.01	Plasma Membrane
**Transport**									
62	A0A0K9RCQ9	Purple acid phosphatase	PAP	FLEEC^5^LASANR	*Spinacia oleracea*	0.40	–	0.03	Extracellular
63	731352863	Probable inactive purple acid phosphatase 29	PAP	QEEVIC^6^PGVNSGFFDTMR	*Beta vulgaris* subsp. vulgaris	0.68	0.75	0.01	Extracellular
64	731320622	Importin subunit alpha	IMP	NATWTLSNFC^10^R	*Beta vulgaris* subsp. vulgaris	1.21	–	0.03	Nucleus
65	A0A061E090	Vaculolar sorting receptor 3 isoform 1	VSR	VC^2^EC^4^PLVDGVQFR	*Theobroma cacao*	0.70	–	0.02	Golgi apparatus
66	731352092	Vacuolar-sorting receptor 4	VSR	YC^2^APDPEQDFSR	*Beta vulgaris* subsp. vulgaris	0.61	–	0.02	Golgi apparatus
67	A0A2N9HVW5	Mitochondrial import receptor subunit TOM40-1-like protein	TOM40	EEEKVDYFNLPC^12^PIPYEEIHR	*Fagus sylvatica*	–	1.81	0.02	Mitochondrion
**Cellular structure**									
68	731336429	Actin-depolymerizing factor	ADP	TGTPAESYDDFLAVLPGNDC^20^R	*Beta vulgaris* subsp. vulgaris	–	0.76	0.01	Extracellular
69	731320854	Actin-depolymerizing factor	ADP	TGGPAESYDDFLASLPESDC^20^R	*Beta vulgaris* subsp. vulgaris	–	0.76	0.02	Extracellular
70	731375712	Basic 7S globulin	Bg7s	TIAPFNVC^8^VDPSTFPASR	*Beta vulgaris* subsp. vulgaris	10.20	5.10	0.04	Plasma Membrane
71	731317399	Profilin-3	Pfn	TGQALVIGLYDEPVTPGQC^19^NMIVER	*Beta vulgaris* subsp. vulgaris	1.29	–	0.03	Cytoplasm
72	A4GDT3	Profilin-1	Pfn	TGQALVFGIYEESVTPGQC^19^NMVVER	*Olea europaea*	1.53	–	0.01	Cytoplasm
73	731354018	Profilin	Pfn	TGQALVFGIYDEPVAPGQC^19^NMVVER	*Beta vulgaris* subsp. vulgaris	1.40	–	0.03	Cytoplasm
**Signal transduction**									
74	731337809	Protein TAPETUM DETERMINANT 1	TPD	C^1^LGFSTVQPVNPR	*Beta vulgaris* subsp. vulgaris	–	1.50	0.01	Plasma Membrane
75	731357482	Ubiquitin domain-containing protein DSK2b	DSK2b	SLVAQNC^7^DVPAEQQR	*Beta vulgaris* subsp. vulgaris	–	0.74	0.05	Nucleus
76	A0A287MC57	Ubiquitin-like domain-containing protein	Uds	LMNAYC^6^DR	*Hordeum vulgare* subsp. vulgare	–	0.80	0.00	Nucleus
77	731354496	Ribosome-inactivating protein PD-L1/PD-L2	Ubls	NQVEAPIRIC^10^GLPSTR	*Beta vulgaris* subsp. vulgaris	2.04	–	0.01	Cytoplasm
78	731345483	Auxin-binding protein ABP19a	ABP	GPEGYAC^7^RDPATLTTDDFVYTGFR	*Beta vulgaris* subsp. vulgaris	0.42	–	0.02	Cell wall
79	A0A2K1KH59	Protein kinase domain-containing protein	AMPK	C^1^IPYLTR	*Physcomitrium patens*	0.76	–	0.04	Cytoplasm
80	731370564	Receptor-like serine/threonine-protein kinase SD1-8 isoform X1	RIPK	TAFVNDGLNLDQC^13^R	*Beta vulgaris* subsp. vulgaris	0.70	–	0.01	Plasma Membrane
81	731348205	Cell wall/vacuolar inhibitor of fructosidase 1	C/VIF1	FGEQAMVDAGNEAEGC^16^R	*Beta vulgaris* subsp. vulgaris	–	1.43	0.02	Vacuole
**Transcription**									
82	731323512	Transcription elongation factor TFIIS	TFIIS	IC^2^NLTAEEMASEQR	*Beta vulgaris* subsp. vulgaris	0.62	–	0.02	Nucleus
83	731358684	Glycine-rich RNA-binding protein	RBP	C^1^FVGGLAWATDDR	*Beta vulgaris* subsp. vulgaris	0.72	–	0.01	Cytoplasm
84	731363127	KH domain-containing protein	KHP	IGETVPGC^8^DER	*Beta vulgaris* subsp. vulgaris	0.76	–	0.03	Nucleus
85	731317968	RNA-binding KH domain-containing protein PEPPER	RBP	VSGVGDVEGSADAAAYC^17^SIR	*Beta vulgaris* subsp. vulgaris	–	1.24	0.04	Nucleus
86	A0A1D1Z0S0	U6 snRNA-associated Sm-like protein LSm7	–	SLGLIVC^7^R	*Anthurium amnicola*	–	1.26	0.00	Nucleus
87	A0A0C9S8X9	Transcribed RNA sequence	–	C^1^GNVNFSFR	*Wollemia nobilis*	1.72	–	0.01	Cytoplasm
**Biosynthesis**									
88	A0A0J8C157	Eukaryotic translation initiation factor 6	eIF6	NC^2^LPDSVVVQR	*Beta vulgaris* subsp. vulgaris	–	0.72	0.02	Nucleus
89	731369461	Eukaryotic translation initiation factor 3 subunit D	eIF3	C^1^ELQSALDINNQR	*Beta vulgaris* subsp. vulgaris	–	1.27	0.03	Cytoplasm
90	1108926884	Elongation factor Tu, chloroplastic	EF-TU	MEVELIHPVAC^11^EEGMR	*Beta vulgaris* subsp. vulgaris	–	0.80	0.03	Cytoplasm
**Photosynthesis**									
91	731341540	Uclacyanin-3-like	–	AQNYVATAVQPC^12^C^13^QGISDAINNER	*Beta vulgaris* subsp. vulgaris	–	0.64	0.00	Plasma Membrane
92	731349464	Ferredoxin, root R-B1	Fd	LIGPDGQVSEFDAPDDC^17^YILDSAENEGVEIPYSC^34^R	*Beta vulgaris* subsp. vulgaris	–	0.61	0.02	Chloroplast
**Unknown**									
93	M1DDJ2	Uncharacterized protein	–	QSHMSLSFSILITELC^16^QR	*Solanum tuberosum*	1.63	–	0.00	Cytoplasm
94	B9T2R9	Clp R domain-containing protein	CLP	INSC^4^ISIEPSLR	*Ricinus communis*	–	1.24	0.01	Cytoplasm
95	M8AU58	Uncharacterized protein	–	MTPTTLAC^8^IGAAAETALPPTHPLR	*Aegilops tauschii*	–	1.53	0.04	Cytoplasm

^a^Protein ID, gi number of NCBI

^b^Sequence with modification, the lower case letter are phosphorylation site in each peptide

^c^iodoTMT salt200/control Ratio, a relative abundance of proteins at redox peptide level (200 mM NaCl treatment versus control), P-value < 0.05

^d^iodoTMT salt400/control Ratio, a relative abundance of proteins at redox peptide level (400 mM NaCl treatment versus control), P-value < 0.05

^e^Protein location, refer to subcellular location prediction website (YLoc, LocTree3, ngLOC, TargetP)

### Functional classification and subcellular localization of root redox proteins

**Fig. 3:**
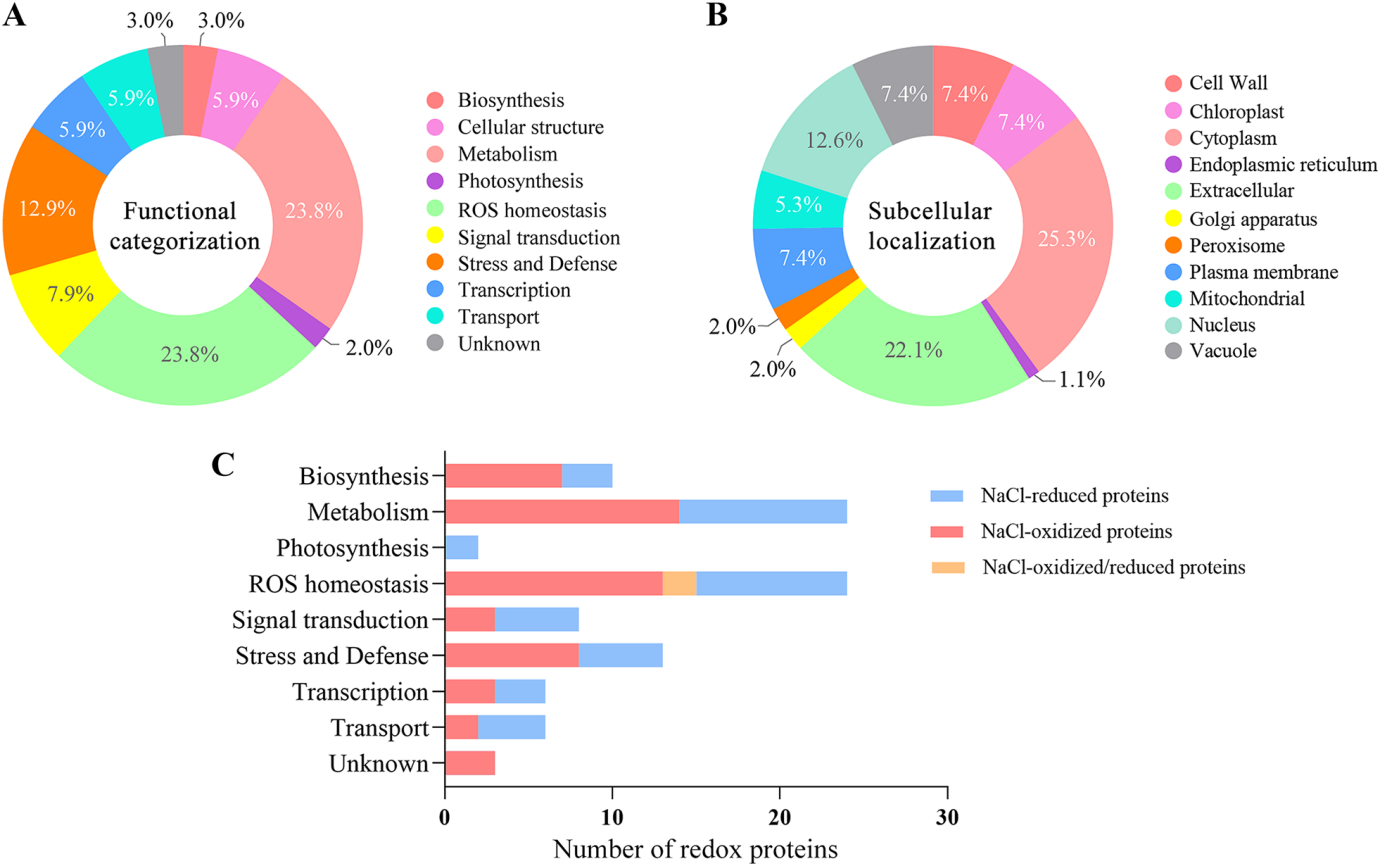
Functional classification and subcellular localization of the differential redox proteins. **A** Functional classification of the differential redox proteins. **B** Subcellular localization prediction of the differential redox proteins. **C** Number of redox proteins in each function

**Fig. 4:**
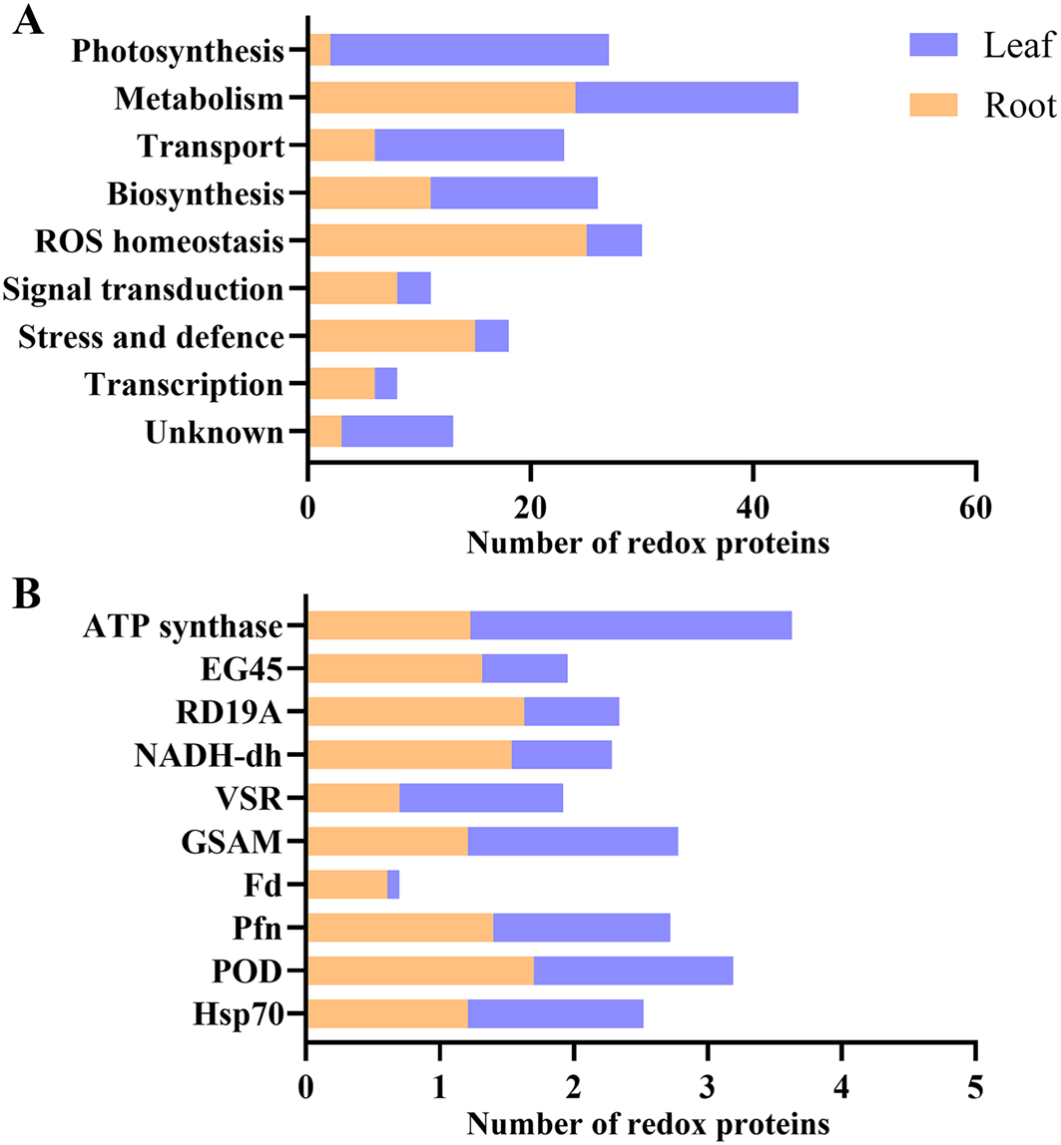
Comparative analysis of differential redox proteins in sugar beet M14 roots and leaves under salt stress. **A** Comparative analysis of redox protein functions under salt stress in roots and leaves. **B** Comparison of protein redox levels under salt stress in roots and leaves of the M14. *EG45* EG45-like domain containing protein, *RD19A* cysteine protease RD19A, *NADH* NADH dehydrogenase [ubiquinone] 1 alpha, *VSR* vacuolar-sorting receptor, *GSAM* glutamate-1-semialdehyde 2,1-aminomutase, *Fd* ferredoxin, root R-B1, *Pfn* profilin, *POD* peroxidase, *Hsp* heat shock cognate protein

The 95 redox proteins under salt stress were divided into nine functional groups (Fig. 3A). A large proportion of redox proteins were involved in the regulation of ROS homeostasis (25.3%), carbohydrate, amino acid and basal metabolism (24.2%), stress and defence (21.1%), and signal transduction (8.4%). A small number of proteins are involved in transport (6%), transcription (6%), photosynthesis (2%), and some proteins are of unknown functions (3%). Subcellular localization showed that the majority of proteins were localized in the cytoplasm (25.3%), extracellular (22.1%), nucleus (12.2%) and others in the cell wall (7.4%), chloroplasts (7.4%), plasma membrane (7.4%) and vacuole (7.4%), mitochondria (5.1%), Golgi apparatus (2%) and peroxisomes (2%) and endoplasmic reticulum (1.1%) (Fig. 3B). We found that more proteins were increased than decreased in oxidative levels in each functional group under salt stress (Fig. 3C). Notably, most of the proteins involved in metabolism and maintenance of ROS homeostasis were oxidized. In contrast, more proteins were reduced or irreversibly oxidized in other processes. Redox proteins are involved in different biological processes in leaves and roots. Unlike roots, leaf redox proteins are mainly involved in photosynthesis, transport, and biosynthesis (Fig. 4A). Such results suggest that the molecular mechanisms of salt resistance in sugar beet M14 roots and leaves are different. GO enrichment results were further analysed in terms of biological processes, molecular function and cellular composition for 95 differential redox proteins (Additional file 1: Fig. S1). The biological processes involved are metabolic process, cellular process, response to stimulus, developmental process, etc. The cellular components were catalytic activity, binding, antioxidant activity, etc. These results suggest that proteins with elevated levels of oxidation in metabolism and maintenance of ROS homeostasis have a dominant role in the tolerance of sugar beet M14 roots to salt stress. In contrast, decrease of protein oxidation levels in other processes was more favorable for salt stress response in sugar beet M14 root systems.

### Transcriptional analysis of differential redox proteins and differential proteins

**Fig. 5:**
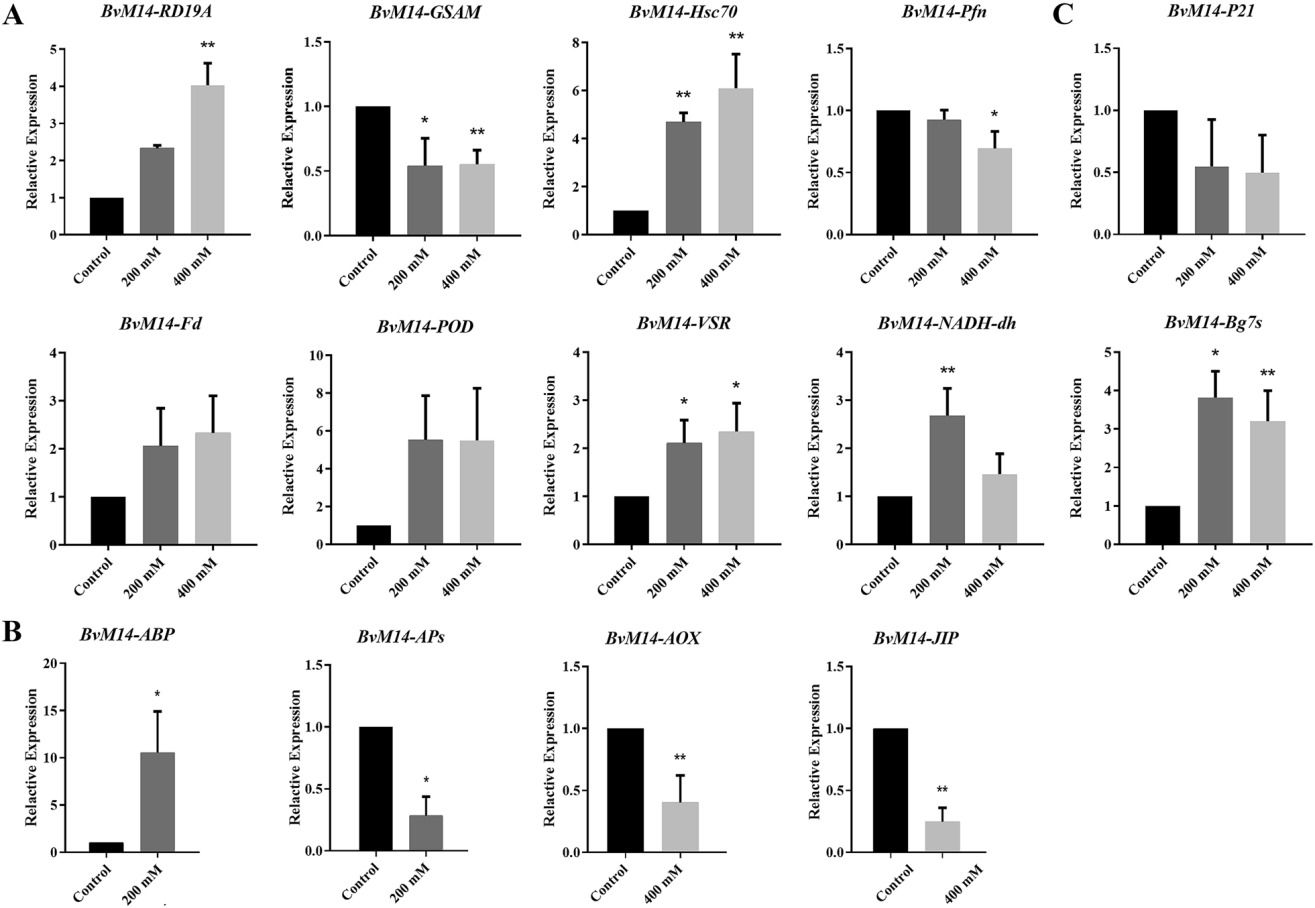
Real-Time PCR assays of genes encoding differential redox proteins and differential proteins in different pathways. **A** RealTime PCR assays of genes encoding redox proteins common to roots and leaves under salt stress, **B** RealTime PCR assays of genes encoding redox proteins specific to 200 mM or 400 mM salt stress condition, and **C** Real-Time PCR assays of genes encoding redox proteins common to 200 mM and 400 mM salt stress. The x-axis is the salt concentration. y-Axis is the relative expression of each gene (2^−ΔΔCT^). Please refer to Table 1 for abbreviations

Key redox proteins were selected for transcript level analysis according to the following criteria. First, we selected three proteins whose oxidation levels were significantly increased after both 200 mM and 400 mM salt stress. Second, proteins specifically involved in maintaining ROS homeostasis, signal transduction, stress and defense regulation and metabolism were selected whose oxidation levels were significantly altered under 200 mM or 400 mM salt stress. Finally, proteins that were identified in both roots and leaves after salt stress, as well as those with significantly altered redox levels, were selected. The expression patterns of these 14 functional genes under salt stress were analyzed by qRT-PCR using the primers in Additional file 6: Table S5. As shown in Fig. 5, of the 14 genes encoding differential proteins, the transcript levels of five genes coincided with the corresponding redox level trends (Additional file 7: Table S6). This suggests that key genes encoding redox proteins can be induced at the transcriptional level by salt stress, and then function through the redox post-translational modifications.

### Overview of potential salt stress response mechanisms in sugar beet M14

**Fig. 6:**
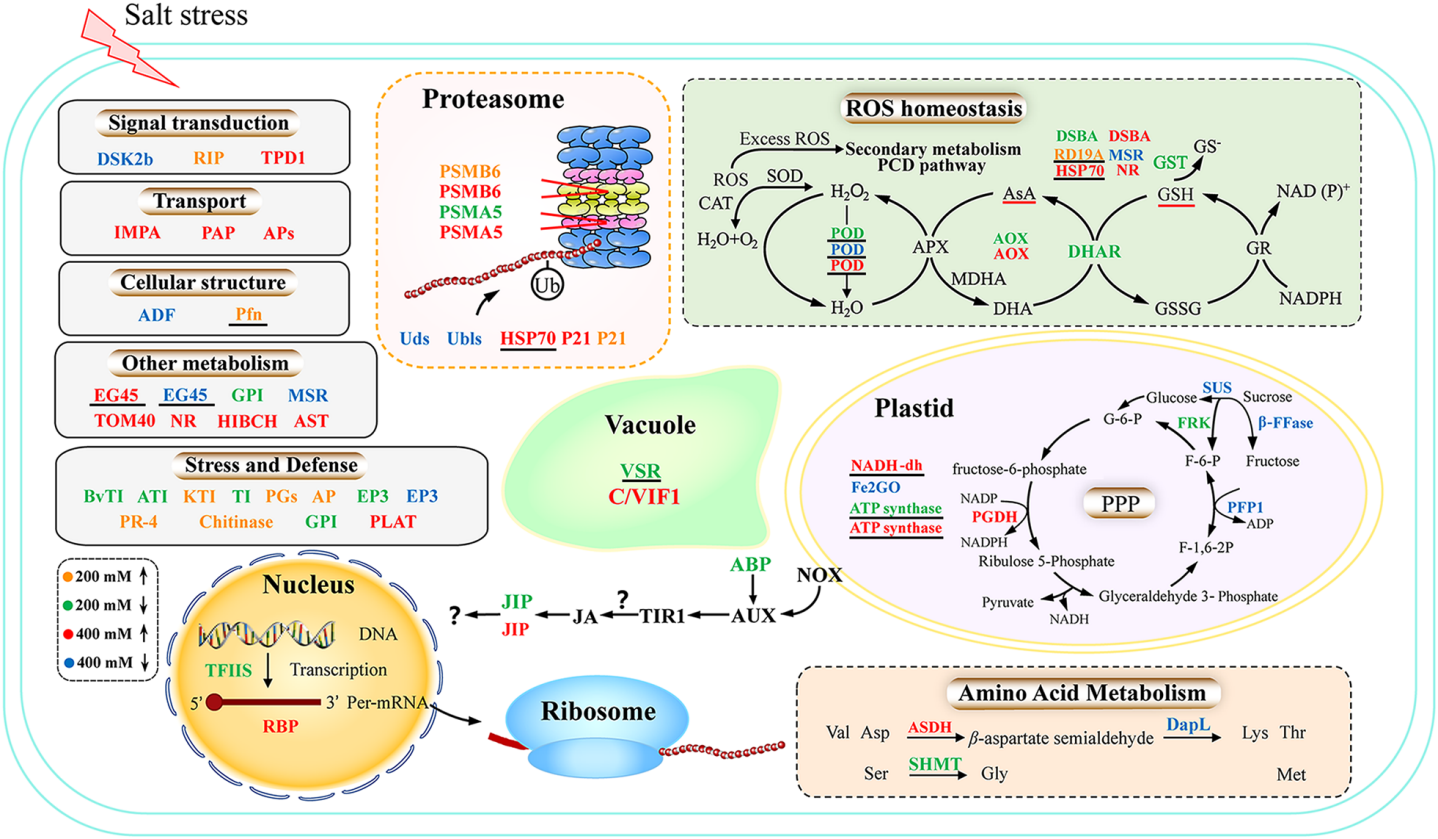
The metabolic networks of the redox protein in sugar beet M14 roots under salt stress. Under 200 mM NaCl treatment, the reduced protein is orange colors and the oxidized protein is green colors. Under 400 mM NaCl treatment, the reduced protein is red colors and the oxidized protein is blue colors. Please refer to Additional file 7: Table S6 for abbreviations. The black underline represents redox proteins common to both leaves and roots

Based on the redox proteomics results including functional classification, KEGG pathway as well as relevant literature, a preliminary network map of redox proteins in response to salt stress in the roots of sugar beet M14 strain was developed (Fig. 6). The redox proteins are marked with yellow and green representing proteins with significantly increased or decreased oxidation levels under 200 mM NaCl treatment. Red and blue represent proteins with significantly increased or decreased oxidation levels under 400 mM NaCl treatment. Plant roots sense salt stress signals and then transmit the signals to the cells via ion signaling and ROS accumulation, leading to oxidative stress. In the roots of sugar beet M14, 25% of the 95 redox proteins identified were involved in maintaining ROS homeostasis, and most of these redox proteins were directly involved in the ROS scavenging process. A small number of redox proteins also provided reducing power to the ROS scavenging system and accelerated the scavenging of ROS in plants under salt stress. In addition, significant changes in the redox levels of protein subunits involved in the ubiquitin–proteasome system were also identified (Fig. 6). Such redox modifications may affect the degradation of oxidatively modified proteins under salt stress, thus contributing to the protein turnover and resistance of plants to salt stress.

## Discussion

Salt stress leads to changes in the levels of PTMs in plants, which regulate the localization, accumulation and activity of proteins. Therefore, studying differential PTM proteins in plants under salt stress will contribute to understanding the complex adaptive mechanisms of plants under adverse environmental conditions. Here we used an iodoTMTRAQ double-labelling approach to study changes in redox modifications of sugar beet M14 root proteins in response to salt stress. Our goal was to compare and contrast the differential redox proteins in sugar beet M14 roots under salt stress with those in the leaves, to ultimately understand sugar beet salt tolerance mechanisms.

### Roots maintain ROS homeostasis through redox modification of antioxidant enzymes and antioxidants

In this study, the number of proteins with increased oxidation was significantly higher in roots of sugar beet M14 under high salt treatment (400 mM NaCl) than that at moderate salt treatment (200 mM NaCl). Some proteins were also found to be almost entirely decreased under the 200 mM salt concentration, while oxidation levels were significantly increased at 400 mM salt. Changes in the oxidation levels of several antioxidant enzymes, including ascorbate oxidase (AOX), dehydroascorbate reductase (DHAR) and peroxidase (POD), were found in the antioxidant system. This caught our attention, and we hypothesize that *BvM14* initiates plant defense mechanisms in extreme environments by regulating protein oxidation levels in roots. It enhances the ROS scavenging capacity of plants, repairs oxidatively modified proteins under salt stress and regulates various metabolic pathways.

AOX and DHAR promote the regeneration of AsA (Yu et al. 2021). AOX catalyzes the oxidation of AsA to dehydroascorbic acid (DHA) via a monodehydroascorbic acid (MDHA) intermediate, which produces AsA following DHAR (Farida et al. 2020). AOX can undergo reversible oxidative modifications and can promote the accumulation of AsA. This could explain the decreased AOX oxidation levels under 200 mM salt stress and the apparently increased oxidation under 400 mM salt stress treatments. The enzymatic activity of DHAR is regulated by reduced sulfhydryl groups in *Arabidopsis* (Tullio et al. 2013). In the present study, Cys12 of DHAR was identified to be decreased in oxidation levels under salt stress. This indicates that the elevated catalytic activity of DHAR is induced under salt stress, which promotes the regeneration of AsA to scavenge ROS in plants and thus improves the tolerance of the *BvM14* roots to salt stress.

**Fig. 7:**
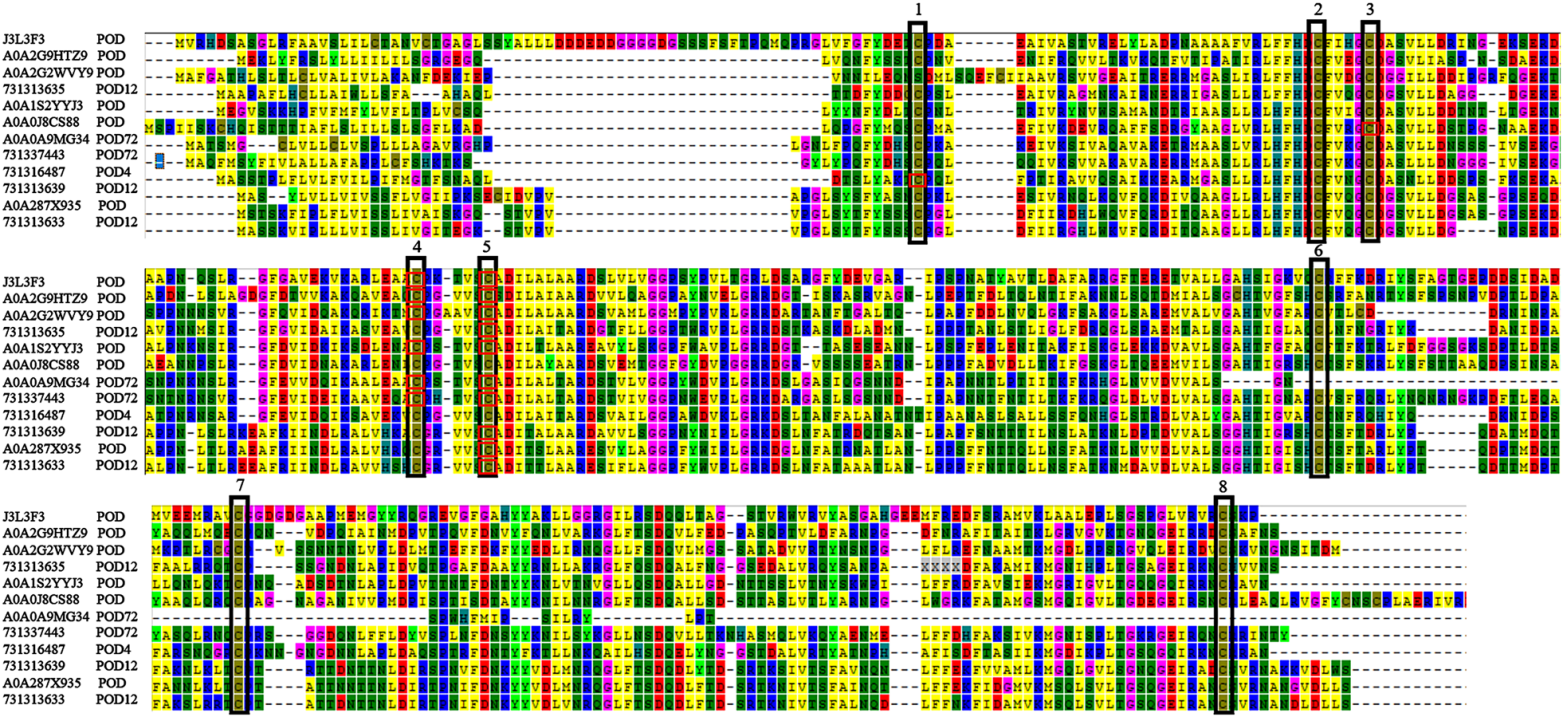
Alignment of amino acid sequence of different expression of peroxidase in salt stress response. Black boxes indicate conserved Cys sites and red boxes indicate Cys sites that undergo redox modifications

The main function of POD is to reduce H_2_O_2_ to H_2_O and to scavenge ROS in plants (Bodra et al. 2017). Salt stress treatment of sugar beet M14 roots revealed altered redox levels of 12 PODs. Further multiple comparisons of amino acid sequences revealed that oxidative modifications occurred at eight conserved Cys sites and were mainly concentrated at two of these Cys sites (Fig. 7). It was found that POD was able to sense the level of ROS based on the oxidation status of Cys (Liu et al. 2014), indicating that the catalytic activity of POD may be induced by high salt stress. The results suggest that changes in the redox status and enzymatic activity of various antioxidant enzymes can regulate and scavenge ROS, which in turn promotes plant tolerance to salt stress.

### Salt stress induces significant changes in protein redox levels in protein degradation systems

The ubiquitin–proteasome system (UPS) is the main pathway for protein degradation in eukaryotic cells (Xu and Xue 2019). Ubiquitin domain-containing protein (Uds) and ubiquitin-like domain-containing protein (Ubls) were decreased at the oxidation level in roots of salt-stressed sugar beet M14. Four proteasomes (three proteasome subunit alpha type-5 and one proteasome subunit beta-6) were identified, three of which had significantly increased oxidation levels. Ubiquitin modified proteins are transported to the proteasome via ubiquitin structural domain proteins, and proteins with ubiquitin tags are recognized by 19S regulatory particles to enter the 26S protease for hydrolysis (Genschik et al. 1994). Redox proteomic findings suggest that the protein degradation system itself may be regulated by redox. How redox and ubiquitination crosstalk in the sugar beet M14 roots to confer salt stress response and tolerance is not known (Harshbarger et al. 2015; Roos and Messens 2011).

### Salt stress affects redox state of proteins in glucose metabolism and amino acid metabolism

Redox proteomics studies have identified significantly increased expression levels of two sucrose synthase isoform (SUS) proteins under salt stress. The SUSs are widely distributed glycosyltransferases in plants and catalyze the catabolism of sucrose. The accumulation of SUS in plant roots under abiotic stresses has been identified several times (Liu et al. 2019; Orlowski et al. 2008; Sasaki et al. 2001; Sharif et al. 2019). SUSs were shown to be involved in osmoregulatory processes, and the sucrose breakdown products promoted cell wall biosynthesis or glycolysis (Albrecht and Mustroph 2003). In this study, SUS oxidation levels were found to be significantly decreased. This suggests that it may act as an osmoregulatory substance to promote plant root tolerance to salt stress by redox activation. In addition, significant changes in the redox levels of four key enzymes [6-phosphogluconate dehydrogenase (PGDH), UDP-glucose 6-dehydrogenase (UGDH), beta-fructofuranosidase, soluble isoenzyme I (FFase) and Pyrophosphate–fructose 6-phosphate 1-phosphotransferase subunit alpha (PFP1)] involved in the sugar metabolism pathway were determined. The redox levels of four enzymes that catalyze aspartate synthesis and metabolism [Aspartate-semialdehyde dehydrogenase (ASDH), Aspartate aminotransferase (AST), Aspartic proteinase A1-like (Aps) and Diaminopimelate aminotransferase (DapL)] were significantly altered, with increased expression of ASDH, AST and Aps. In subsequent studies, the glucose and aspartate contents in the roots of sugar beet M14 strain could be measured to further verify the effects of redox modifications on the activities of key enzymes in the sugar and amino acid metabolism pathways.

### Relationship between redox proteins and phosphorylation-modified proteins

Protein phosphorylation modifications are one of the most fundamental and important post-translational modifications. In eukaryotes, phosphorylation modifications occur mainly on residues of serine, threonine and tyrosine. Hsp70 binds to nascent polypeptides on the ribosome, inhibiting the process of folding newly synthesised proteins (Beckmann et al. 1990). It also acts as a molecular chaperone to carry proteins, transporting them to different cellular compartments (Getting and Sambrook 1992). Under moderate salt stress, Hsp70 was phosphorylated. At high concentrations of salt stress, the phosphorylation level of Hsp70 was down-regulated while the oxidation level was up-regulated. This may indicate that Hsp70 plays different roles in signal transduction or other pathways under different levels of salt stress. Notably, phosphorylated proteins are inextricably linked to the regulation of intracellular kinases and phosphatases and are involved in a variety of cellular processes, such as transmembrane or intracellular signaling, conformation change of proteins, and subcellular trafficking (Hsu et al. 2009; Jørgensen and Linding 2008; Zhou et al. 2018). For example, it was found that the phosphorylation of the Ser534 site of Arabidopsis nitrate reductase (NR) is sensitive to exogenous H_2_O_2_. Interestingly, the Met538 site of NR acts as a recognition element for Ser534 phosphorylation. The Met538 site is oxidized to methionine sulfoxide (MetSO), and this redox modification oxidation significantly inhibits the phosphorylation modification of the Ser534 site. Coupling redox signal to changes in protein phosphorylation is important (Hardin et al. 2009). In addition, we identified AMPK, a protein with multiple phosphorylation sites, which plays a key role in the regulation of anabolism or catabolism by directly phosphorylating proteins or by regulating gene transcription in various pathways such as lipid synthesis, oxidation and lipolysis (Wang et al. 2018). Receptor-like serine-/threonine-protein kinase (RSTK) was decreased at phosphorylation levels and significantly increased at oxidation levels in previous studies (Tyler and Friedman 2004; Wang et al. 2014; Yu et al. 2016). RSTK belongs to the receptor-like kinase (rlk/pelle) family. Rlk/pelle family proteins can interact with other proteins and play an important signal role in pathogen recognition, activation of plant defense mechanisms and developmental regulation (Li et al. 2002). RSTK may contribute to the tolerance of sugar beet M14 lines to salt stress by regulating the levels of redox and phosphorylation modifications, while the effect of oxidation on phosphorylation levels needs to be further investigated.

### Different strategies employed in salt stress responses in roots and leaves of sugar beet M14

Under salt stress, signals are sensed by the cell membrane and transmitted to organelles such as chloroplasts, mitochondria and the nucleus in plant leaves (Fig. 6). Redox levels of proteins involved in photosynthesis are significantly altered and play a dominant role in salt stress. The leaves regulate the redox levels of photosynthesis-related proteins and influence protein conformation, thereby regulating protein function to ensure that plants receive the energy they need to survive salt stress. Unusually, roots accelerate the rate of ROS scavenging and maintain ROS homeostasis in plants under salt stress, mainly through significant changes in the redox levels of antioxidant enzymes and related proteins that provide reducing power to the ROS scavenging system, thereby improving the salt tolerance. Ten redox proteins from leaves and roots were found to respond synergistically to salt stress (Fig. 4B). Among them, the oxidation levels of POD and Hsp70 were significantly increased, while VSR, Fd and GPI were significantly decreased. VSR is a transmembrane receptor protein involved in the targeted transport of soluble vesicular proteins to the vesicle (Kang and Hwang 2014; Soares et al. 2019). In leaves, Fd is the major protein involved in the last step of the photosynthetic electron transport reaction (Hanke et al. 2004). However, Fd is mainly reduced under non-photosynthetic conditions in roots, allowing the reduced Fd state to transfer electrons to NADP+, and the resulting NADPH reducing power may be used in roots or transported to leaves for carbon fixation in the Calvin cycle and other metabolic processes in the chloroplasts. In addition, the reduced state of Fd can also use electrons for other reactions such as nitrogen assimilation, sulphur assimilation, lipid and chlorophyll synthesis, and it also participates in metabolic processes such as the AsA-GSH cycle, thus indirectly regulating ROS homeostasis (Hanke et al. 2004). LysM-GPI was identified in the secretome of grapes in response to cyclodextrin and methyl jasmonate, but the role of LysM-GPI in plant is not known. The specific functions of LysM-GPI in plant resistance pathways have not been reported and need to be further investigated.

## Conclusions

In this study, the root redox proteomics of sugar beet M14 seedlings under salt stress was analysed using iodoTMTRAQ double-labelling technique combined with LC–MS/MS proteomics. A total of 95 redox proteins exhibiting different redox levels were identified. These proteins were involved in metabolism, ROS homeostasis, stress and defense, transport, cell structure, protein folding and degradation, signal transduction, transcription, photosynthesis and some unknown functions. It is clear that while the potential salt response mechanisms involve many different components, pathways and processes, root redox proteins are central to those involved in the regulation of ROS homeostasis (Fig. 6). Interestingly, crosstalk between redox and phosphorylation was noted. Subcellular localization predictions showed that most redox proteins were predicted to be localized in the cytoplasm and extracellular compartments. Combined analysis of the differential redox proteins in M14 leaves, we can achieve a comprehensive understanding of the mechanisms of post-translational modifications under salt stress in the special *BvM14*, which is conducive to a profound analysis of the salt tolerance mechanism in sugar beet. Real-time PCR of genes encoding 14 important redox proteins showed that four proteins had consistent expression at the transcript level and protein level. Based on the experimental results, a working model to guide future functional studies was proposed for the potential involvement of redox proteins and phosphoproteins in response to salt stress in the roots of sugar beet M14.

## Supplementary Information

**Additional file 1: Figure S1.** Singular enrichment analysis (SEA) for redox proteins in biological process (A), cellular components (B) and molecular function (C) was conducted using AgriGO. Each box shows the GO term, GO description, the number mapping the GO and total number of query in the background. Box color indicates levels of statistical significance. More statistically significant nodes result in darker red color.

**Additional file 2: Table S1.** List of the identified 2305 proteins in *BvM14* roots under control and 200 mM NaCl treatment using LC–MS/MS.

**Additional file 3: Table S2.** List of the identified 2663 proteins in *BvM14* roots under control and 400 mM NaCl treatment using LC–MS/MS.

**Additional file 4: Table S3.** List of redox proteins identified using iodoTMTRAQ in *BvM14* roots under control and 200 mM NaCl treatment.

**Additional file 5: Table S4.** List of redox proteins identified using iodoTMTRAQ in *BvM14* roots under control and 400 mM NaCl treatment.

**Additional file 6: Table S5.** List of the primer sequences for the 14 genes tested by qRT-PCR in Fig. 5.

**Additional file 7: Table S6.** The transcriptional level, redox protein level of 14 differential redox proteins.

## Data Availability

The data and materials used and analyzed in the current study can be provided by the corresponding author for scientific, non-profit purposes.
