# Supplementary figures and images for: Quantitative redox proteomics revealed molecular mechanisms of salt tolerance in the roots of sugar beet monomeric addition line M14
Source: Bot Stud. 2022 Mar 5;63:5. doi: 10.1186/s40529-022-00337-w (PMC8898211; doi:10.1186/s40529-022-00337-w)

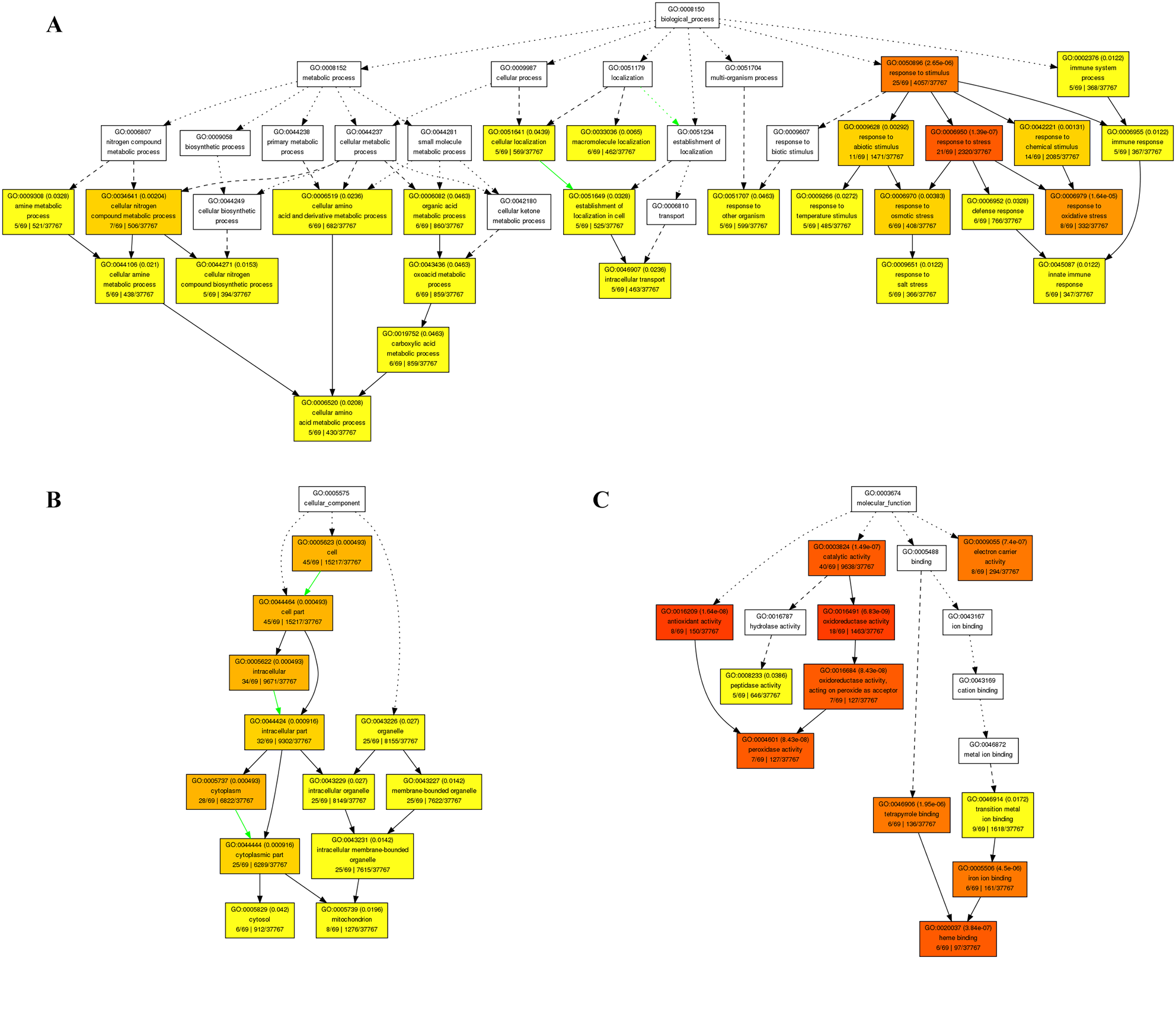

Supplement: Supplementary file 1 — Additional file 1: Figure S1. Singular enrichment analysis (SEA) for redox proteins in biological process (A), cellular components (B) and molecular function (C) was conducted using AgriGO. Each box shows the GO term, GO description, the number mapping the GO and total number of query in the background. Box color indicates levels of statistical significance. More statistically significant nodes result in darker red color. [file 40529_2022_337_MOESM1_ESM.tif]
