# Supplementary material for: Quantitative redox proteomics revealed molecular mechanisms of salt tolerance in the roots of sugar beet monomeric addition line M14
Source: Bot Stud. 2022 Mar 5;63:5. doi: 10.1186/s40529-022-00337-w (PMC8898211; doi:10.1186/s40529-022-00337-w)
Supplement: Supplementary file 6 — Additional file 6: Table S5. List of the primer sequences for the 14 genes tested by qRT-PCR in Fig. 5. [file 40529_2022_337_MOESM6_ESM.docx]

Table S5. List of the primer sequences for the 14 genes tested by qRT-PCR in Figure 5.

| **No** | **Protein ID^a^** | **Gene name^b^** | **Primer name** | **Primer sequences^c^** | |
| --- | --- | --- | --- | --- | --- |
| 1 | 731312054 | *BvM14-RD19A* | RD19A-S | | 5’-actcatgtgactctggctgc-3’ |
|  |  |  | RD19A -AS | | 5’-tggtcttcatcaagggcgac-3’ |
| 2 | A0A0K9RN52 | *BvM14-GSAM* | GSAM-S | | 5’- cttcactgatgggcctgtgt-3’ |
|  |  |  | GSAM-AS | | 5’- gctgctgctatcgttcgttg-3’ |
| 3 | A0A166FTZ6 | *BvM14-Hsp70* | Hsp70-S | | 5’-ttgtcaagacgatcgcacca-3’ |
|  |  |  | Hsp70-AS | | 5’-ttgtcaagacgatcgcacca-3’ |
| 4 | 731354018 | *BvM14-Pfn* | Pfn-S | | 5’-ggaagcgtttgggctcaaag-3’ |
|  |  |  | Pfn-AS | | 5’-cgaacactagagcttggcca-3’ |
| 5 | 731349464 | *BvM14-Fd* | Fd-S | | 5’-ccccggatgactgctacatc-3’ |
|  |  |  | Fd-AS | | 5’-accgctgtccatttgaccat-3’ |
| 6 | A0A0J8CS88 | *BvM14-POD* | POD-S | | 5’-taaacgtcaaggcctcgacc-3’ |
|  |  |  | POD-AS | | 5’-cgcaactgagaagcatacgc-3’ |
| 7 | 731352092 | *BvM14-VSR* | VSR-S | | 5’- aatgccaatgtcctccaggg-3’ |
|  |  |  | VSR-AS | | 5’- tacattcagggcactggcag-3’ |
| 8 | 731357289 | *BvM14-NADH-dh* | NADH-dh-S | | 5’-tcccacatcggcggttttaa-3’ |
|  |  |  | NADH-dh-AS | | 5’-tcctgctctttgcggcataa-3’ |
| 9 | 731345483 | *BvM14-ABP* | ABP19a-S | | 5’-tggcggtaacttacctgctg-3’ |
|  |  |  | ABP19a-AS | | 5’-agtgccaaatgccctcttca-3’ |
| 10 | 731353768 | *BvM14-APs* | APs-S | | 5’-agcaactcggggtctagact-3’ |
|  |  |  | Aps-AS | | 5’-gcaaatccctggtaggcact-3’ |
| 11 | 731355863 | *BvM14-AOX* | AOX-S | | 5’-atacctcacgctttggtgca-3’ |
|  |  |  | AOX-AS | | 5’-gaactgcccattgatcccca-3’ |
| 12 | 731326017 | *BvM14-JIP* | JIP-S | | 5’-tggcggtaacttacctgctg-3’ |
|  |  |  | JIP-AS | | 5’-agtgccaaatgccctcttca-3’ |
| 13 | 731331163 | *BvM14-P21* | P21-S | | 5’-ttccacttggactgtcaccg-3’ |
|  |  |  | P21-AS | | 5’-ctctactgcacccacctgtg-3’ |
| 14 | 731375712 | *BvM14-Bg7s* | Bg7s-S | | 5’-ttgtcaagacgatcgcacca-3’ |
|  |  |  | Bg7s-AS | | 5’-atgtccatgcttgactcgca-3’ |
| 15 |  | *BvM14-rRNA^d^* | 18S-S | | 5’-CCCCAATGGATCCTCGTTA-3’ |
|  |  |  | 18S-FS | | 5’-TGACGGAGAATTAGGGTTCG-3’ |

^a^ Protein ID, gi number of NCBI.

^b^ Gene name, the genes of 14 differential redox proteins.

^c^ Primer sequence, the primer sequences for 14 genes (s represents sense primer of the gene, as represents anti-sense primer of the gene).

*^d^ 18S rRNA*, the reference gene for real-time PCR in sugar beet M14.
