# Supplementary material for: Quantitative redox proteomics revealed molecular mechanisms of salt tolerance in the roots of sugar beet monomeric addition line M14
Source: Bot Stud. 2022 Mar 5;63:5. doi: 10.1186/s40529-022-00337-w (PMC8898211; doi:10.1186/s40529-022-00337-w)
Supplement: Supplementary file 7 — Additional file 7: Table S6. The transcriptional level, redox protein level of 14 differential redox proteins. [file 40529_2022_337_MOESM7_ESM.docx]

Table S6. The transcriptional level, redox protein level of 14 differential redox proteins.

| **No.** | **Protein ID^a^** | **Gene name^b^** | **qRT-PCR**  **(200mM**  **/control^c^)** | **qRT-PCR**  **(400mM**  **/control^d^)** | **Protein name^e^** | **Redox level (200mM**  **/control^f^)** | **Redox level (400mM /control^g^)** |
| --- | --- | --- | --- | --- | --- | --- | --- |
| 1 | 731312054 | *BvM14-RD19A* | **↑** | **↑** | *BvM14*-RD19A | **↑** | **––** |
| 2 | A0A0K9RN52 | *BvM14-GSAM* | **↓** | **↓** | *BvM14*-GSAM | **↑** | **—** |
| 3 | A0A166FTZ6 | *BvM14-Hsp70* | **↑** | **↑** | *BvM14*-Hsp70 | **––** | **↑** |
| 4 | 731354018 | *BvM14-Pfn* | **↓** | **↓** | *BvM14*-Pfn | **↑** | **—** |
| 5 | 731349464 | *BvM14-Fd* | **↑** | **↑** | *BvM14*-Fd | **—** | **↓** |
| 6 | A0A0J8CS88 | *BvM14-POD* | **↑** | **↑** | *BvM14*-POD | **––** | **↑** |
| 7 | 731352092 | *BvM14-VSR* | **↑** | **↑** | *BvM14*-VSR | **↓** | **—** |
| 8 | 731357289 | *BvM14-NADH-dh* | **↑** | **↑** | *BvM14*-NADH-dh | **—** | **↑** |
| 9 | 731345483 | *BvM14-ABP* | **↑** | **—** | *BvM14*-ABP | **↓** | **—** |
| 10 | 731353768 | *BvM14-APs* | **↓** | **—** | *BvM14*-APs | **—** | **↑** |
| 11 | 731355863 | *BvM14-AOX* | **––** | **↓** | *BvM14*-AOX | **↓** | **↑** |
| 12 | 731326017 | *BvM14-JIP* | **––** | **↓** | *BvM14*-JIP | **—** | **↑** |
| 13 | 731331163 | *BvM14-P21* | **↓** | **↓** | *BvM14*-P21 | **↑** | **↑** |
| 14 | 731375712 | *BvM14-Bg7s* | **↑** | **↑** | *BvM14*-Bg7s | **↑** | **↑** |

^a^ Protein ID, gi number of NCBI.

^b^ Gene name, the genes of 14 differential redox proteins.

^c^ RT-PCR (200mM/control), the transcriptional level of the gene between 200mM salt treatment and control.

^d^ RT-PCR (400mM/control), the transcriptional level of the gene between 400mM salt treatment and control.

^e^ Protein name, the genes of 14 differential redox proteins.

^f^ redox level (200mM/control), the ratio of redox peptide between 200mM salt treatment and control.

^g^ redox level (400mM/control), the ratio of redox peptide between 400mM salt treatment and control.

––, no change or redox modification. ↓, downregulated expression. ↑, upregulated expression.
